# Endothelial Cells Promote Migration of Mesenchymal Stem Cells via PDGF-BB/PDGFR*β*-Src-Akt in the Context of Inflammatory Microenvironment upon Bone Defect

**DOI:** 10.1155/2022/2401693

**Published:** 2022-09-24

**Authors:** Sihao He, Tianyong Hou, Jiangling Zhou, Qiuchi Ai, Ce Dou, Fei Luo, Jianzhong Xu, Junchao Xing

**Affiliations:** ^1^National Regional United Engineering Laboratory of Tissue Engineering, Department of Orthopedics, Southwest Hospital, Army Military Medical University, Chongqing, China; ^2^Center of Regenerative and Reconstructive Engineering Technology in Chongqing City, Chongqing, China; ^3^Tissue Engineering Laboratory of Chongqing City, Chongqing, China

## Abstract

Homing of mesenchymal stem cells (MSCs) to the defect site is indispensable for bone repair. Local endothelial cells (ECs) can recruit MSCs; however, the mechanism remains unclear, especially in the context of the inflammatory microenvironment. This study was aimed to investigate the role of ECs in MSCs migration during the inflammatory phase of bone repair. The inflammatory microenvironment was mimicked *in vitro* via adding a cytokine set (IL-1*β*, IL-6, and TNF-*α*) to the culture medium of ECs. The production of PDGF-BB from ECs was measured by ELISA. Transwell and wound healing assays were employed to assess MSCs migration toward ECs and evaluate the implication of PDGF-BB/PDGFR*β*. A series of shRNA and pathway inhibitors were used to screen signal molecules downstream of PDGF-BB/PDGFR*β*. Then, mouse models of femoral defects were fabricated and DBM scaffolds were implanted. GFP^+^ MSCs were injected via tail vein, and the relevance of PDGF-BB/PDGFR*β*, as well as screened signal molecules, in cell homing was further verified during the early phase of bone repair. In the mimicked inflammatory microenvironment, MSCs migration toward ECs was significantly promoted, which could be abrogated by *pdgfrb* knockout in MSCs. Inhibition of Src or Akt led to negative effects analogous to *pdgfrb* knockout. Blockade of JNK, MEK, and p38 MAPK had no impact. Meanwhile, the secretion of PDGF-BB from ECs was evidently motivated by the inflammatory microenvironment. Adding recombinant PDGF-BB protein to the culture medium of ECs phenocopied the inflammatory microenvironment with regard to attracting MSCs, which was abolished by *pdgfb*, *src*, or *akt* in MSCs. Moreover, *pdgfb* knockout suppressed the expression and phosphorylation of Src and Akt in migrating MSCs. *Src* knockout impaired Akt expression but not vice versa. *In vivo*, reduced infiltration of CD31^+^ ECs was correlated with diminished PDGF-BB in local defect sites, and silencing *pdgfb*, *src*, or *akt* in MSCs markedly hampered cell homing. Together, these findings suggest that in the inflammatory microenvironment, MSCs migrate toward ECs via PDGF-BB/PDGFR*β* and the downstream Src-Akt signal pathway.

## 1. Introduction

Large segmental bone defects caused by trauma, tumor resection, or bone infection remain among the most prevalent clinical challenges. The *in situ* tissue engineering concept, which is based on attracting and modulating osteoprogenitors already present in a patient's body to accelerate bone repair, has become a promising strategy. The reparative specialty of resident mesenchymal stem cells (MSCs) is worthless unless their directional homing is appropriately controlled [1]. Physiologically, MSCs reside in the bone marrow niche, and their engagement and disengagement maintain a dynamic balance. Upon injury, an inflammatory microenvironment is triggered locally and the balance is disrupted, leading to abundant cell egression into circulation and migration to the injury sites [2]. Meanwhile, local vascularization is reinforced in response to inflammation. During bone repair, angiogenesis, the formation of new blood vessels from preexisting ones, is closely coupled with osteogenesis [3]. To a certain extent, the onset time and extent of revascularization determine the outcome of bone grafting [4]. Therefore, the inflammatory microenvironment and vascularization situation are critical factors for effective MSCs homing. Nevertheless, the precise functional modes and mechanisms remain confused.

During the embryonic and postnatal periods, intimate physical proximity exists between blood vessels and osteoprogenitors, implying a close relationship between endothelial cells (ECs) and MSCs [5]. Indeed, the crosstalk of ECs and MSCs has been widely documented and exploited to ameliorate blood supply and expedite tissue regeneration [6]. Initially, attention was given to the transdifferentiation of MSCs into ECs or the regulatory effects of MSCs on ECs [7]. For example, we and others have reported that MSCs can attract endothelial lineages via the chemokine-receptor cascade reactions [8, 9]. Currently, there is growing interest in the inversus effects. Recent findings suggest that a certain subset of ECs, mainly referred to as Type-H ECs (CD31^hi^Emcn^hi^), precedes and guides homing of osteoprogenitors [3]. Reduction of EC infiltration by VEGFR2 antagonist impedes migration of osteoprogenitors and bone reconstruction. ECs can facilitate tissue regeneration through their paracrine capacity [10], by which they communicate with osteoprogenitors. The secretome of ECs is influenced by the local microenvironment, including inflammation. Among EC-derived biologics, platelet-derived growth factor (PDGF) family is famous for regulating the viability and proliferation of MSCs [11]. Thereinto, the PDGF-BB homodimer appears to be highly potent in fostering osteogenesis via activation of multiple kinase-dependent signaling cascades [12]. Besides, the ability of PDGF-BB in promoting cell migration has been widely documented [13]. The major receptor, platelet-derived growth factor receptor-*β* (PDGFR*β*), is believed to mobilize cells of mesenchymal origin [13].

Here, we tried to investigate the chemotactic effects of ECs on MSCs in the early inflammatory microenvironment following bone defects, as well as the involvement of PDGF-BB/PDGFR*β*, aiming to shed light on the early angio-osteogenic coupling and provide therapeutic targets for *in situ* bone tissue engineering. First, the inflammatory microenvironment was mimicked *in vitro* as reported previously [14]. Next, MSCs movement toward ECs was evaluated via migration assays. The relevance of PDGF-BB/PDGFR*β* was defined via gene silence and pathway inhibitors. Then, signal molecules downstream of PDGF-BB/PDGFR*β* were screened out *in vitro* and further validated *in vivo*. Eventually, we concluded that in the inflammatory microenvironment, ECs promoted MSCs migration via the PDGF-BB/PDGFR*β*-Src-Akt pathway.

## 2. Materials and Methods

### 2.1. Cell Culture

All experiments on human and animal samples were approved by the Ethics Committee, Southwest Hospital, Army Military Medical University. Human bone marrow MSCs (hBMSCs) and human umbilical vein endothelial cells (HUVECs) were purchased from Cyagen Biosciences (HUXMA-01001, HUVEC-20001). hBMSCs were cultured in basic culture medium containing Dulbecco's modified Eagle's medium/F12 (DMEM/F12; 1 : 1; HyClone, USA) supplemented with 10% fetal bovine serum (FBS; Gibco, USA) and 100 U/ml penicillin/streptomycin (Gibco, USA). HUVECs were cultured in Endothelial Cell Growth Medium-2 (EGM; CC-3162; Lonza, Switzerland) containing 10% FBS and 100 U/ml penicillin/streptomycin. The media were changed every other day. When reaching 80-90% confluence, cells were digested using 0.25% trypsin-EDTA (Gibco, USA) and passaged. HUVECs at passage 3 and hBMSCs at passage 4 were harvested for use.

Mouse mesenchymal stem cells (mBMSCs) were isolated and cultured as described previously [9]. Briefly, bone marrows were extracted from femurs by resecting the epiphyses and flushing the shaft with cold phosphate buffered saline (PBS; Beyotime, China). Cells were collected by centrifugation and resuspended in basic culture medium containing DMEM/F12 supplemented with 15% FBS and 100 U/ml penicillin/streptomycin. Then, cells were incubated in a 5%-CO_2_ incubator at 37°C. After 24 h, nonadherent cells were discarded and the culture media were changed every 48-72 h. When reaching confluence of more than 80%, cells were trypsinized and passaged for 3 times before use.

### 2.2. Preparation of Conditioned Media

HUVECs were incubated with EGM added with or without 4 ng/ml IL-1*β*, 10 ng/ml IL-6, and 20 ng/ml TNF-a (all from PeproTech, Rocky Hill, NJ, USA) to prepare conditioned media of inflammatory ECs (IEC-CM) or ECs (EC-CM). After 48 h, the supernatants were collected, centrifuged, aliquoted, and stored at -80°C. EGM free of serum and supplemented with stromal cell-derived factor 1 (SDF-1; 100 ng/ml) served as negative and positive controls, respectively. The contents of PDGF-BB in IEC-CM and EC-CM were measured using a human ELISA kit (Solarbio, Beijing, China) according to the manufacturer's instructions.

### 2.3. Gene Interference

HUVECs, hBMSCs, and GFP^+^mBMSCs (obtained from GFP transgenic C57 mice) were infected with lentivirus particles encoding the corresponding short hairpin RNA (shRNA; Santa Cruz Biotechnology, Dallas, TX, USA) according to the manufacturer's instructions. Clones expressing the virus were selected by their resistance to puromycin (Sigma-Aldrich, St. Louis, Missouri, USA). The interference efficiency was confirmed by western blot.

### 2.4. Migration Assay

Transwell inserts with 8-mm pores (Corning, NY, USA) were used for *in vitro* migration assays. Conditioned medium (700 ml) was added to the bottom compartment. hBMSCs were pre-treated with serum-free medium or medium supplemented with inhibitors, as detailed in Table 1. The upper chamber of Transwell insert was filled with 5 × 10^4^ cells, which were allowed to migrate at 37°C. Cells in partial groups were collected for biochemical analysis at 30 min. After 8 h, hBMSCs on the top side of the insert (non-migrating cells) were dislodged with a cotton tip applicator. Then, the migrated cells on the bottom side were washed with PBS, followed by fixation with 4% paraformaldehyde (Boster Biologic Technology, Wuhan, China). The membrane was moved onto one object slide with the lower side upward. Cells retained on the membrane were labeled with DAPI (Invitrogen, Carlsbad, CA, USA) and subjected to fluorescent microscopy. For every group, three high-power fields (HPF, ×200) were randomly chosen and migrated hBMSCs were counted and averaged. Migration assay was repeated in triplicate.

For the wound healing assay, groups of hBMSCs were seeded and cultured in 6-well plates (1 × 10^5^/well) to reach the confluent monolayer. Then, cells were scraped using a 200 *μ*L pipette tip and washed with PBS to clear cell debris and suspension. Complete medium was replaced with conditioned media and cells were incubated for 12 h. Microscopic images were captured at the same position of the wound at 0 and 12 h. Migration ability was measured by the rates of scratch wound closure using the ImageJ software (National Institutes of Health, Maryland, USA).

### 2.5. RT-PCR

Primers are shown in Table 2. Total RNA was extracted using TRIzol reagents (Invitrogen, Carlsbad, CA). cDNA was prepared using a cDNA Synthesis Kit (TaKaRa, Japan) and RT-PCR was implemented with a QuantiTect™SYBR Green PCR Kit (TaKaRa, Japan). Glyceraldehyde 3-phosphate dehydrogenase (GAPDH) served as a reference control. Reactions were repeated in triplicate.

### 2.6. Western Blot

SDS lysis buffer (100 mM Tris at pH 8.0, 10% glycerol, and 1% SDS) was used for cell lysis. Using a NanoVue spectrophotometer (GE Healthcare, Waukesha, WI, USA), the protein concentration was measured. For each sample, protein lysate (30 mg) was isolated by SDS-PAGE (120 min, 80 V; Beyotime, Shanghai, China) and electrotransferred to the polyvinylidene fluoride membrane (60 min, 250 mA; Millipore, MA, USA). After blocking using milk (5%), each membrane was incubated at 4°C for 12 h with the corresponding primary antibodies, which are detailed in Table 3. Following a thorough wash with TBST, the blots were incubated with the secondary antibody (horseradish peroxidase-conjugated, 1 : 2000; Southern Biotech, AL, USA) at room temperature (RT) for about 1 h. The membranes were visualized by ECL (Kirkegaard&Perry Lab, MD, USA). GAPDH was used as control. All experiments were repeated for 3 times.

### 2.7. Animal Manipulation

Decalcified bone matrices (DBM) were prepared from the trabecular bones of Yunnan miniature pigs and surgeries were performed according to procedures reported previously [15]. Femoral critical-sized bone defects (2 mm in length) were created in C57 mice (8weeks, male) and DBM were implanted. Implants harvested at postoperative days 1, 3, and 7 were subjected to fluorescence-activated cell sorting (FACs), RT-PCR, and western blot. At 7 days, wild GFP^+^ mBMSCs or cells intervened by shRNA were injected (1 × 10^6^/mouse) via tail vein every 2 days (Supplemental Figure 1) [16, 17].

### 2.8. FACs

Cells were harvested from implants by sufficient digestion, which was achieved by using Type I collagenase (1 mg/ml) and trypsin (0.25%) plus EDTA (0.01%; Thermo Fisher Scientific, MA, USA). Then, cells in each group were filtered and centrifuged. After resuspension in PBS containing 2% FBS, cells were incubated with the antibody against CD31 (fluorescence-conjugated; BD Biosciences, CA, USA) at 4°C for 30 min. Non-stained cells were incubated with the isotype control. After centrifugation and resuspension in propidium iodide, the sample was subjected to a CytoFLEX S Flow Cytometer (Beckman Coulter, CA, USA). Data were inspected with the CytoFlex software. For each group, the experiment was repeated in triplicate.

### 2.9. Immunofluorescent Staining

Three mice from each group were euthanized. The femurs were collected, fixed with paraformaldehyde (4%) for 24 h, and decalcificated using EDTA for 7-10 days. Then, 7-mm-thick frozen sections were prepared and permeabilized using Triton X-100 (0.3%), followed by blocking with donkey serum (1 : 20; Huayueyang Biotech, Beijing, China). Subject slides were incubated with primary antibodies (Table 3) at 4°C for 12 h. Then, samples were stained with the corresponding secondary antibodies (Table 3) for 1 h and counterstained with DAPI for about 10 min. Randomly, three separate sections were selected from more than 20 sections for each group. Relative cellularity was measured by counting stained cells in five HPF using a confocal laser scan microscope (Leica Biosystems, Wetzlar, Germany).

### 2.10. Histological Observation

At 4 weeks postoperatively, the implants were obstained, decalcified with EDTA (10%), dehydrated in graded alcoholic solutions, and embedded in paraffin. 7-mm-thick sections were prepared and stained using by hematoxylin and eosin (HE) and Masson's trichrome methods. Using a microscope (Olympus, Hamburg, German), images were taken.

### 2.11. Statistical Analysis

Data were presented as means±SEM. For ELISA, RT-PCR, western blot, FACs, and migration assay, one-way ANOVA followed by SNK test was conducted to determine the statistical significance between groups (SPSS v.13.0). The correlation analysis was identified by Pearson's correlation coefficient. A value of *P* < 0.05 was considered statistically significant.

## 3. Results

### 3.1. ECs Promote MSCs Migration in the Inflammatory Microenvironment

**Figure 1:**
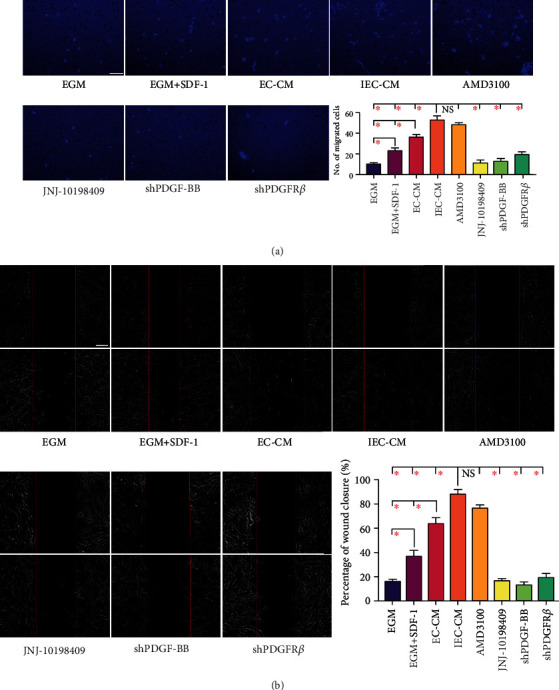
MSCs migrated toward ECs via PDGF-BB/PDGFR*β* in the inflammatory microenvironment. (a) Representative images of migrated hBMSCs that had received different pre-treatments or had been exposed to different inducing media. The migration capacity of hBMSCs was determined using a Transwell culture system. The quantification of migrated cells was shown as a bar graph. Scale bar, 50 *μ*m. ∗*P* < 0.05. (b) Representative images of wound healing assays. The rate of scratch wound closure was shown as a bar graph. Scale bar, 200 *μ*m. ∗*P* < 0.05. EGM: endothelial cell growth medium-2. EC-CM: conditioned media of endothelial cells. IEC-CM: conditioned media of endothelial cells in the context of inflammatory microenvironment; shPDGF-BB: short hairpin RNA targeting *pdgfb*; shPDGFR*β*: shRNA targeting *pdgfrb*.

Compared with control, EC-CM and IEC-CM significantly promoted MSCs migration, and IEC-CM showed the strongest chemotactic power (Figure 1(a)). SDF-1 and its receptor CXCR4 have been widely documented to be indispensable for MSCs migration. Yet, the chemotactic power of EC-CM and IEC-CM was much greater than that of SDF-1(Figure 1(a)). Meanwhile, AMD3100, a specific CXCR4 antagonist, had no effect on IEC-CM-induced migration. Consistent findings were obtained from the wound healing assay (Figure 1(b)). Although both EC-CM and SDF-1 induced MSCs migration, IEC-CM showed the highest rate of scratch area closure. Together, these findings reinforced the recruiting effect of ECs on MSCs, which could be remarkably enhanced by the inflammatory microenvironment.

### 3.2. MSCs Migrate toward ECs via PDGF-BB/PDGFR*β* in the Inflammatory Microenvironment

**Figure 2:**
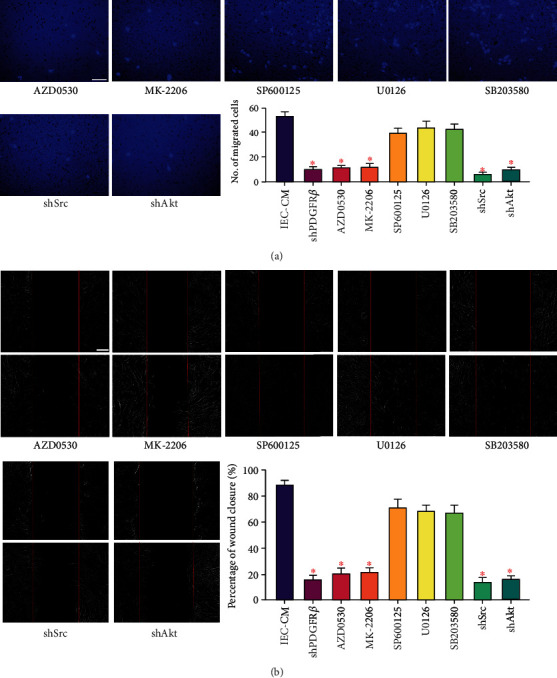
Src and Akt were required for ECs-induced MSCs migration in inflammatory microenvironment. (a) Representative images of migrated hBMSCs in Transwell culture systems. Pathway inhibitors were used for pre-treating cells migrating to IEC-CM. The quantification of migrated cells was shown as a bar graph. Data were compared with the groups of IEC-CM and shPDGFR*β* from Figure 1. Scale bar, 50 *μ*m. ∗*P* < 0.05. (b) Representative images of wound healing assays. The rate of scratch wound closure was shown as a bar graph. Data were compared with the groups of IEC-CM and shPDGFR*β* from Figure 1. Scale bar, 200 *μ*m. ∗*P* < 0.05. IEC-CM: conditioned media of endothelial cells in the context of inflammatory microenvironment; shPDGFR*β*: short hairpin RNA targeting *pdgfrb*; shSrc: shRNA targeting *src*; shAkt: shRNA targeting *akt*.

Other than SDF-1/CXCR4, signaling pathways mediated by PDGF-BB/PDGFR*β* possess a crucial role in stem cell motility. To unveil the mechanism underlying MSCs migration toward ECs in the inflammatory microenvironment, PDGFR*β* was firstly blocked in MSCs using a specific inhibitor, JNJ-10198409. MSCs migration toward IEC-CM was abrogated by JNJ-10198409 (Figure 2(a)), underlining the relevance of PDGFR*β*. Then, the concentration of PDGF-BB in the conditioned media was measured. ECs secreted PDGF-BB spontaneously, and the inflammatory microenvironment memorably forced the production (Supplemental Figure 2A). Thereafter, genes of *pdgfb* and *pdgfrb* were knocked out by shRNA in cultured ECs and MSCs, respectively. The interference efficiencies of shRNA were checked by western blot. Sh-1 for PDGF-BB and sh-2 for PDGFR*β* were chosen for use (Supplemental Figure 2B, 2C). As a result, MSCs migration toward IEC-CM was dramatically impeded by shRNA targeting PDGF-BB in ECs or PDGFR*β* in MSCs (Figure 1(a)). Furthermore, the wound healing assay verified the reduced ability of MSCs in repairing the damaged area after gene silence (Figure 1(b)). These results collectively suggested that ECs-induced MSCs migration in the inflammatory microenvironment was attributed to the activation of PDGF-BB/PDGFR*β*.

### 3.3. Src and Akt Function Downstream of PDGFR*β* during MSCs Migration toward ECs

**Figure 3:**
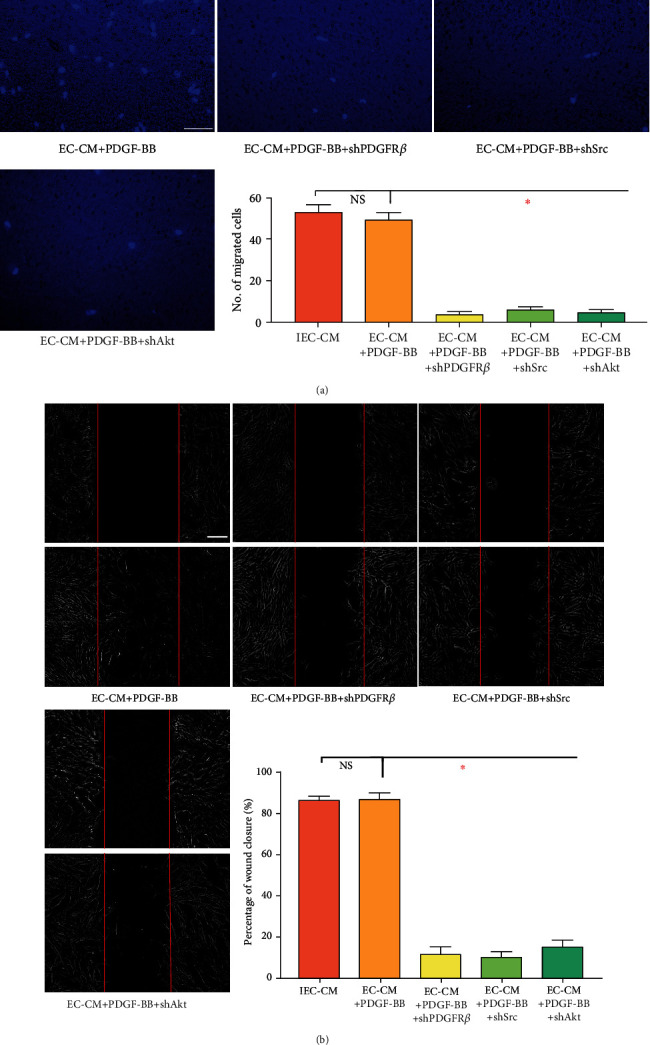
Src and Akt functioned downstream of PDGFR*β*. (a) Representative images of migrated hBMSCs in Transwell culture systems. The quantification of migrated cells was shown as a bar graph. Data were compared with the group of IEC-CM from Figure 1. Scale bar, 50 *μ*m. ∗*P* < 0.05. (b) Representative images of wound healing assays. The rate of scratch wound closure was shown as a bar graph. Data were compared with the group of IEC-CM from Figure 1. Scale bar, 200 *μ*m. ∗*P* < 0.05. IEC-CM: conditioned media of endothelial cells in the context of inflammatory microenvironment; shPDGFR*β*: short hairpin RNA targeting *pdgfrb*; shSrc: shRNA targeting *src*; shAkt: shRNA targeting *akt*.

In terms of cell migration, various signaling molecules have been identified to be associated with PDGF-BB/PDGFR*β*, such as phosphoinositide 3-kinase (PI3K)/protein kinase B (Akt), c-JunN-terminal kinase (JNK), mitogen-activated protein (MEK), mitogen-activated protein kinase (MAPK), and steroid receptor coactivator (Src). Next, we tried to screen signals downstream of PDGFR*β*, based on a series of highly selective pathway inhibitors. Accordingly, pre-treating MSCs with an inhibitor of JNK (SP600125), MEK (U0126), or p38 MAPK (SB203580) only slightly weakened migration toward IEC-CM (Figure 2(a)). In contrast, AZD0530 (Src) or MK-2206 (Akt) led to a remarkable migratory energy in a manner similar to *pdgfrb* interference, indicating the implication of Src and Akt. Then, genes of *src* and *akt* were knocked out in MSCs (Supplemental Figure 2C) and markedly suppressed cell migration toward IEC-CM (Figure 2(a)). Analogical findings were obtained from the wound healing assay (Figure 2(b)). MSC movement to the scratch region, induced by IEC-CM, was significantly inhibited after knockout of *pdgfrb*, *src*, or *akt*. Nevertheless, blockade of JNK, MEK, or p38 MAPK showed no obvious difference. To figure out the relationship of Src and Akt with PDGF-BB, recombinant PDGF-BB protein was added to EC-CM. PDGF-BB elevated the chemotctic power of EC-CM to a level similar to that of IEC-CM (Figure 3). The augmentative effect of PDGF-BB was abrogated by knockout of *pdgfrb*, *src*, or *akt* in MSCs. Collectively, these findings suggested that Src and Akt were effectors downstream of PDGF-BB/PDGFR*β*.

**Figure 4:**
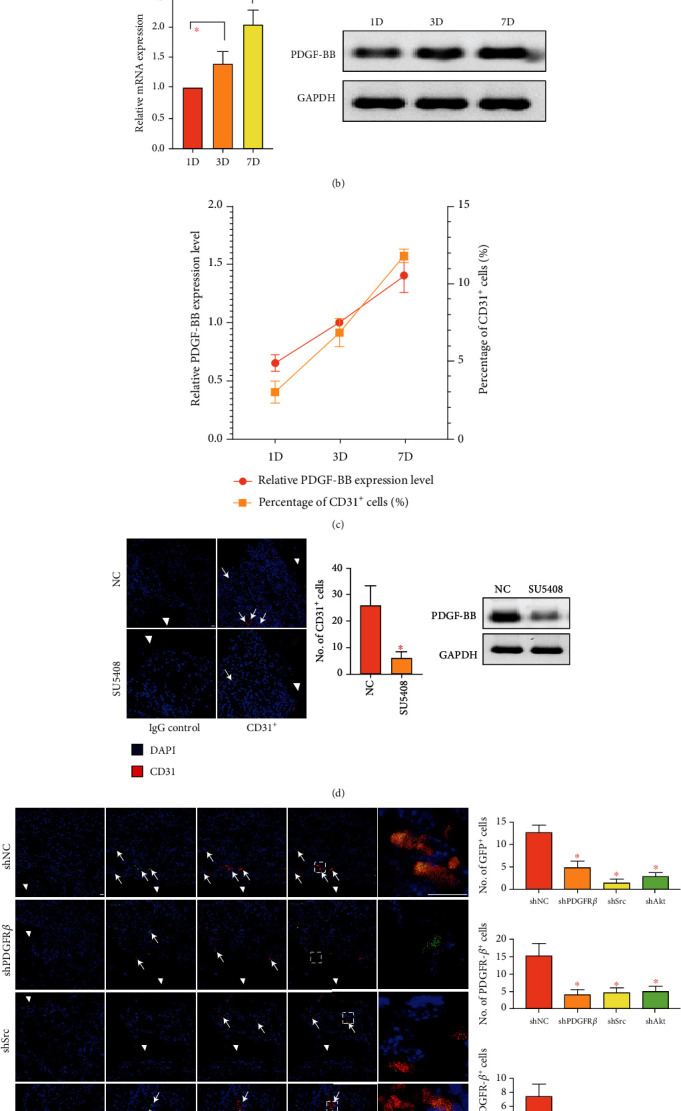
Data of *in vivo* experiments. (a) Fluorescence-activated cell sorting of CD31^+^ cells within implants at postoperative days 1, 3, and 7. The percent of CD31^+^ cells migrated cells was shown as a bar graph. (b) Gene and protein expression of PDGF-BB in sorted CD31^+^ cells. Data were shown as bar graphs. (c) A positive correlation lay between the ratio of CD31^+^cells and the protein level of PDGF-BB (Pearson's correlation coefficient *R* =0.926, *P* < 0.05). (d) Representative images of infiltrated CD31^+^ cells and PDGF-BB levels within implants. The quantitative comparison was detailed as bar graphs (*n* =5). (e) Representative images of homed MSCs within implants. The quantitative comparisons were detailed as bar graphs (*n* =5). Scale bars, 10 *μ*m. ∗*P* < 0.05. Triangles, implants. Arrows, staining positive cells. shPDGFR*β*: short hairpin RNA targeting *pdgfrb*; shSrc: shRNA targeting *src*; shAkt: shRNA targeting *akt*.

For further verification, *in vivo* experiments were performed. CD31^+^ ECs that infiltrated in the implantation area were sorted and the expression of PDGF-BB was detected. The ratio of ECs gradually increased over time (Figure 4(a)). Similar variation trend was gained in the mRNA and protein expression of PDGF-BB (Figure 4(b)). Moreover, a positive correlation existed between the ratio of CD31^+^ ECs and the protein level of PDGF-BB (Pearson's correlation coefficient *R* =0.926, *P* < 0.05; Figure 4(c)). At 7 days, administration of the VEGFR2 specific inhibitor, SU5408, significantly impeded the infiltration of CD31^+^ ECs within implants (Figure 4(d)). In consequence, the concentration of PDGF-BB within implants was sharply reduced to an extremely low level. According to the current literature, VEGF-mediated activation of VEGFR2 suppressed PDGFR*β* signaling in vascular smooth muscle cells through the assembly of the receptor complex consisting of VEGFR2 and PDGFR*β* [18]. Limited by the accessible evidence, the potential effect of SU5408 on the expression of PDGF-BB could not be entirely excluded. Considering that ECs are one of the main sources of PDGF-BB, these findings indirectly suggested that at least in the early inflammatory phase (<7 days), the infiltrated ECs within implants served as an important source of PDGF-BB at local sites. Then, GFP^+^ mBMSCs with gene interference (*pdgfb*, *src*, or *akt*, Supplemental Figure 2D) were administrated via tail intravenous injection. At 10 days, GFP^+^ cells appeared in the graft area and almost all of them expressed PDGFR*β* in the control group (Figure 4(e)). In contrast, knockout of *pdgfb*, *src*, or *akt* resulted in a dramatic decrease in the number of GFP^+^ cells within implants. It was notable that although the amount was small, GFP^+^ cells were present after *pdgfb* knockout but they seldom expressed PDGFR*β*. This might be ascribed to the fact that the interference efficiency of PDGFR*β* was not 100% and the participation of other pathways guiding MSCs homing. Intriguingly, GFP^+^ cells were almost invisible after *src* knockout, but PDGFR*β*^+^ cells were evident, indicating the predominant role of Src in MSCs homing. As compared with control, a smaller number of GFP^+^ cells were observed after *akt* knockout and most of them were PDGFR*β* positive (Figure 4(c)). This finding suggested that despite its crucial roles, Akt might not be indispensable in PDGFR*β*-mediated cell motility as compared with Src. At 4 weeks postoperatively, the healing effects of different treatments for bone defects were compared. As shown in Supplemental Figure 3, bony development was advanced within control group, as the implants were surrounded by chondrocyte, osteoblast-like cells and filled with livable osteocytes. In the other groups, no viable osteocytes were found in lacunas within bone pieces, and implants were poorly embedded by osteogenesis-related cells. These findings indicated the roles of PDGFR*β*, Src, and Akt in the development of bone grafts, thus indirectly providing support for their relevance in motility of host osteoprogenitors.

### 3.4. Src Is a Bridge Connection between PDGFR*β* and Akt during ECs-Induced MSCs Migration

**Figure 5:**
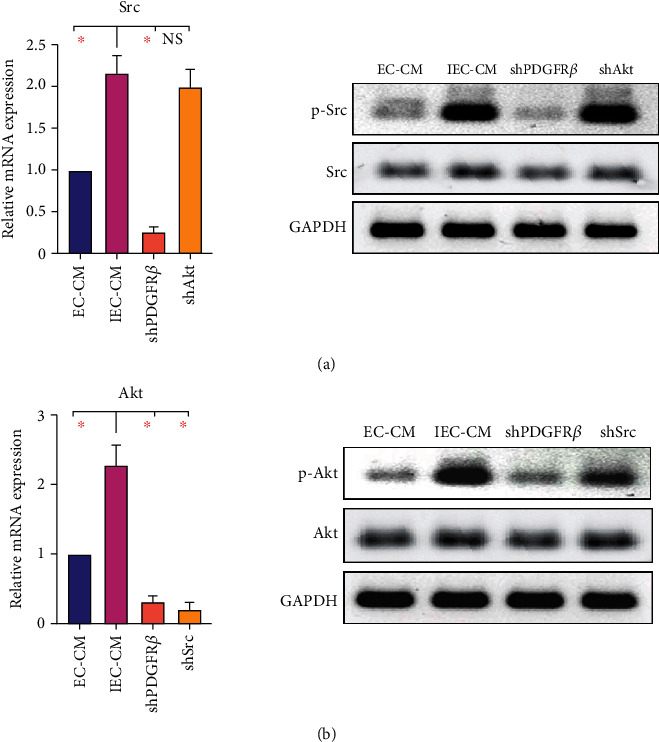
Src bridged connection between PDGFR*β* and Akt during ECs-induced MSCs migration. (a) Gene and protein expression of Src in migrating hBMSCs. (b) Gene and protein expression of Akt in migrating hBMSCs. EC-CM: conditioned media of ECs; IEC-CM: conditioned media of ECs in the context of inflammatory microenvironment; shPDGFR*β*: short hairpin RNA targeting *pdgfrb*; shSrc: shRNA targeting *src*; shAkt: shRNA targeting *akt*. ∗*P* < 0.05.

To reveal the relationship between Src and Akt, migrating MSCs were collected *in vitro*. IEC-CM memorably increased the mRNA expression and phosphorylation of Src in MSCs, which were significantly attenuated by knockout of *pdgfrb*, but not *akt* (Figure 5). Moreover, the mRNA expression and phosphorylation of Akt were elevated by IEC-CM. Notably, knockout of *pdgfrb* or *src* impaired the positive effect of IEC-CM on Akt, although no difference was found in the protein level of total Akt. Based on the findings mentioned above, we concluded that in the inflammatory environment, ECs-induced MSCs migration via the PDGFR*β*-Src-Akt pathway.

## 4. Discussion

For bone repair, angiogenesis and osteogenesis are essential processes taking ECs and MSCs as representative involved cells, respectively. They are closely related as evidence indicates the vicinal spatiotemporal loci between ECs and MSCs during bone development and regeneration [3]. Indeed, many perivascular cells exhibit characteristics of mesenchymal progenitors and possess multilineage differentiation potential [19]. Lineage tracing studies suggest that Nestin expressing cells on arteries represent early mesenchymal stem and progenitor cells, with the potential to generate bone lineage cells [20]. In this context, various types of EC-MSC coculture experiments have been conducted to investigate the mechanism and impact of their crosstalk, especially in the development of bone substitutes. In general, findings are positive as MSCs promote ECs-mediated angiogenesis and ECs may regulate the migration and differentiation of MSCs [21]. Accordingly, their coculture has been widely employed to ameliorate repairing efficacy via forming vasculature and inducing vessel ingrowth prior to and after bone grafting, respectively [6]. Nevertheless, unlike the impact of MSCs on ECs, which has been widely described, less is known on the inverse effects. Besides, most of *in vitro* studies on their crosstalk are performed under normal conditions [9]. However, the influence of cell crosstalk *in vivo* may be entirely different due to the intricate internal environment. Upon bone injury, local inflammatory responses are incited to form a microenvironment rife with bioactive cellular and molecular components [22]. Consequently, angiogenic or osteogenic cells are educated to change secretome and tropism [14]. On one side, the stimulation of angiogenic cells enables and impulses angiogenesis. Other than nutrient supply and metabolite exchange, homing of osteoprogenitors, as well as the fateful event of bone repair, depends heavily on local vascularization status. On the other side, the inflammatory microenvironment modulates the secretome of MSCs and fosters congeneric recruitment [14]. Therefore, the inflammatory situation cannot be ignored while studying EC-MSC crosstalk. In this study, we introduced a mimicking inflammatory microenvironment *in vitro* to investigate cell motility. IL-1*β*, IL-6, and TNF-a are representative pro-inflammatory cytokines with peak levels in the early inflammatory phase of bone healing and play crucial roles in bone reconstruction by triggering highly complicated biological cascades [23]. Compared with common ECs inflammation models, which are usually induced by lipopolysaccharide, the present inflammatory microenvironment is more biomimetic since ECs are exposed during the early stage and cell apoptosis caused by LPS can be avoided effectively [12]. Based on this model, we echoed the concept that ECs could induce MSCs migration physiologically. Under an inflammatory microenvironment, ECs showed a more intensive chemotactic effect on MSCs. This phenomenon is readily comprehensible considering that when new blood vessels grow into the local inflammatory loci, ECs release abundant chemokines to guide vessel-associated MSCs entering to form sheets of osteoblasts, which then secrete osteoid to fabricate bones as oriented by the invading vessels.

Among the multiple chemokines of ECs, the PDGF family has been recognized with significance in the angio-osteogenic coupling [13]. PDGF consists of four polypeptides A, B, C, and D, which assemble into disulfide-linked homodimers or heterodimers (PDGF-AA, -BB, -CC, -DD, or -AB). Thereinto, PDGF-BB is the only dimer with high affinity to all known receptor isoforms and has drawn extensive attention. During bone repair, PDGF-BB plays an integral role in coordinating and linking ECs, MSCs, the extracellular matrix, and signaling pathways [24]. More accurately, PDGF-BB/PDGFR*β* constitutes the principal pathway responsible for the activation and function of MSCs, the proliferation and migration of pericytes, and the development of vasculature and new bones. PDGF-BB is mainly secreted from ECs, preosteoclasts, and platelets and supports migration, proliferation, and differentiation of various bone marrow-derived mesenchymal cells to promote angiogenesis and osteogenesis [13]. Herein, we reported that the inflammatory microenvironment forced ECs to secrete an exponential amount of PDGF-BB. Moreover, the promigratory effect of ECs was visibly inhibited by blockade of PDGFR*β*. These results collectively confirmed the authority of PDGF-BB/PDGFR*β* in osteoprogenitor homing, a pivotal event in the early inflammatory stage of bone repair.

Various signal molecules downstream of PDGF-BB/PDGFR*β* have been identified with influences in different disease models. During bone modeling and remodeling, the binding of PDGF-BB and PDGFR*β* triggered PI3K/Akt and MAPK signaling cascades, promoting the formation of Type-H vessels and the migration of osteoprogenitors [25]. Also, PI3K and MAPK were requisite in PDGF-BB-mediated MSC motility toward glioma [26]. Previous studies on osteogenic MC3T3-E1 cells showed that the mitogenic response stimulated by PDGF-BB was dependent on extracellular signal-regulated kinase (Erk) and JNK, whereas the migratory response involved MAPK and JNK [27]. JNK was further verified with significance in PDGF-induced proliferation and migration of MSCs [28]. Endothelial progenitor cells were reported to facilitate viability and nerve regenerative ability of MSCs via PDGF-BB/PDGFR*β* and downstream PI3K/Akt and MEK/Erk pathways. Besides, Src played key roles in the migration of metanephric mesenchymal cells toward PDGF-BB [29]. This study adopted a set of pathway inhibitors to screen the predominant signal molecules involved. We found that Src and Akt were the main effectors downstream of PDGF-BB/PDGFR*β* during MSC migration toward ECs in the inflammatory microenvironment. Meanwhile, homing of MSCs to bone defects was significantly impaired when *pdgfrb*, *src*, or *akt* was knocked down. Conversely, the results denied the implication of JNK, MEK, and MAPK. With regard to the difference, there were two aspects of conceivable interpretations. One was the extensive regulatory roles of PDGF-BB/PDGFR*β* in cell behaviors: viability, proliferation, differentiation, apoptosis, migration, and communication [24]. Another reason lay in the distinct cell and disease types among the currently available literature [30]. Nevertheless, the concurrent involvement of Src and Akt in PDGF-BB-mediated MSCs migration in the inflammatory microenvironment was verified for the first time.

Although Src and Akt link a variety of cell receptors to elicit impacts, their relationship in terms of cell motility remains confused. Most opinions support the upper position of Src. For example, Src acts upstream of Akt in the neural cell adhesion molecule-regulated proliferation, apoptosis, autophagy, migration, and epithelial-to-mesenchymal transition of human melanoma cells [31]. During ASAP1-regulated osteogenic differentiation of MSCs, Src and Akt were implicated and Akt served as the downstream effector. Yet, there is evidence demonstrating the regulatory effect of Akt on Src [32]. As with MSCs, the influence of Src or Akt has been generally accepted; however, little is known on their interaction with regard to motility regulation. Limited evidence suggests that Src may regulate the proliferation and osteogenic differentiation of MSCs via Akt [33]. Here, we showed that shRNA targeting Src downregulated the mRNA expression and phosphorylation of Akt in MSCs. Conversely, Akt shRNA had no significant effect on Src. Thus, ECs recruited MSCs in the inflammatory environment through PDGF-BB/PDGFR*β* and its downstream Src-Akt signaling pathway.

There are some limitations in the present study. First, we failed to establish the bone defect models in mice where PDGF-BB was conditionally knocked out in ECs. The death rate was excessive after femoral defects were made with the approach detailed above. This indirectly supported the vital roles of PDGF-BB in sustaining ECs function. As a compromise, MSCs with silenced gene expression were injected back via tail vein, making the confidence level a little weak. Second, the secretory profile of ECs in the inflammatory environment was not fully plotted and there may be other biologics and signaling pathways affecting MSCs migration. Finally, the functional mechanism between and following Src and Akt was not assessed in depth. Further experiments based on proteomics and genomics are needed to gain more insights.

In conclusion, to our knowledge, this study first reveals the role of PDGF-BB/PDGFR*β*, as well as downstream Src and Akt signaling, in promoting MSC migration toward ECs in the inflammatory microenvironment. Understanding the functional interplay between ECs and MSCs is practically significant with regard to monitoring processes implicated in bone development after implantation and providing clues for efficacy promotion.

## Figures and Tables

**Table 1:** Reagent information.

Reagent	Target	Concentration	Duration	Usage	Source
AMD3100	CXCR4	5 *μ*g/ml	30 min	Pre-treat MSCs	Sigma-Aldrich, St. Louis, MO, USA
AZD0530	Src	10 *μ*M	24 h	Pre-treat MSCs	Selleck Chemicals, Houston, TX, USA
MK-2206	Akt1/2/3	10 *μ*M	24 h	Pre-treat MSCs	Selleck Chemicals, Houston, TX, USA
U0126	MEK1/2	50 *μ*M	24 h	Pre-treat MSCs	Selleck Chemicals, Houston, TX, USA
SP600125	JNK1/2/3	10 *μ*M	24 h	Pre-treat MSCs	Selleck Chemicals, Houston, TX, USA
SB203580	p38 MAPK	1 *μ*M	30 min	Pre-treat MSCs	Selleck Chemicals, Houston, TX, USA
JNJ-10198409	PDGFR*β*	5 *μ*M	1 h	Pre-treat MSCs	MedChemExpress, Monmouth Junction, NJ, USA
SU5408	VEGFR2	5 *μ*M	1/2d since implantation	Intraperitoneal injection	MedChemExpress, Monmouth Junction, NJ, USA

**Table 2:** Primers for RT-PCR.

Gene	Species	Forward (5′-3′)	Reverse (5′-3′)
Src	Human	AAGCTGAGGCATGAGAAG	GTACTCCGTGACGATGTAA
Akt	Human	TATTGTGAAGGAGGGTTG	ATTCTTGAGGAGGAAGTAG
GAPDH	Human	ATCAACTCACCGCCAACA	CGACTCAATCTTCCTCTCCAG
PDGF-BB	Mouse	CATCGAGCCAAG ACACCTCA	AGTGCCTTCTTGTCA TGGGT
GAPDH	Mouse	CGGATTTGGTCGTATTGG	TCCTGGAAGATGGTGATG

**Table 3:** Antibody information.

Antibody	Usage	Host-reactivity	Dilutions	Clonality	Source
PDGF-BB	Western blot	Rabbit anti-mouse	1 : 500	Polyclonal	Abcam, Cambridge, UK
PDGF-BB	Western blot	Chicken anti-human	1 : 500	Polyclonal	Abcam, Cambridge, UK
PDGFR*β*	Western blot	Rabbit anti-mouse/human	1 : 1000	Monoclonal	Cell Signaling Technology, Danvers, MA, USA
Src	Western blot	Rabbit anti-mouse/human	1 : 1000	Monoclonal	Cell Signaling Technology, Danvers, MA, USA
p-Src	Western blot	Rabbit anti-mouse/human	1 : 1000	Monoclonal	Cell Signaling Technology, Danvers, MA, USA
Akt	Western blot	Rabbit anti-mouse/human	1 : 1000	Monoclonal	Cell Signaling Technology, Danvers, MA, USA
p-Akt	Western blot	Rabbit anti-mouse/human	1 : 2000	Monoclonal	Cell Signaling Technology, Danvers, MA, USA
CD31	Immunofluorescence	Goat anti-mouse	1 : 100	Polyclonal	R&D Systems, Minneapolis, MN, USA
PDGFR*β*	Immunofluorescence	Goat anti-mouse	1 : 100	Polyclonal	R&D Systems, Minneapolis, MN, USA
Anti-GFP	Immunofluorescence	Rabbit anti-GFP	1 : 200	Polyclonal	Abcam, Cambridge, UK
Alexa Fluor® 488	Immunofluorescence	Goat anti-rabbit	1 : 200	Polyclonal	Abcam, Cambridge, UK
NL557	Immunofluorescence	Donkey anti-goat	1 : 500	Polyclonal	R&D Systems, Minneapolis, MN, USA

## Data Availability

The FACs, transwell assay, wound healing assay, immunofluorescent staining, RT-PCR, and western blot data used to support this study's findings are included in the article. The western blot and Elisa data used to support the findings of this study are included within the supplementary information file.

## Supplementary Materials

Supplementary Materials Supplementary Figure 1. Scheme illustration for *in vivo* experiments. Femoral critical-sized bone defects were created in C57 mice and DBM were implanted. Implants harvested at postoperative day 1, 3, and 7 were subjected to fluorescence-activated cell sorting (FACs), RT-PCR, and western blot. At 7 days, wild GFP^+^ mBMSCs or cells intervened by shRNA were injected via tail vein every 2 days. Implants harvested at 10 days and 4 weeks were subjected to immunofluorescence and HE&Masson staining, respectively. Supplementary Figure 2. (A) ELISA results. (B) Interference efficiency of shRNA targeting *pdgfb* in HUVECs. (C) Interference efficiencies of shRNA targeting *pdgfrb*, *src*, and *akt* in hBMSCs. (D) Interference efficiencies of shRNA targeting *pdgfrb*, *src*, and *akt* in mBMSCs. Supplementary Figure 3. Representative images of H&E staining and Masson staining. At 4 weeks postoperatively, bony development was advanced within control group, as the implants were surrounded by chondrocyte, osteoblast-like cells, and filled with livable osteocytes. In the other groups, no viable osteocytes were found in lacunas within bone pieces, and implants were poorly embedded by osteogenesis-related cells. Scale bars, 500 *μ*m.
