# Correction to “Endothelial Cells Promote Migration of Mesenchymal Stem Cells via PDGF‐BB/PDGFRβ‐Src‐Akt in the Context of Inflammatory Microenvironment upon Bone Defect”

**DOI:** 10.1155/sci/9783731

**Published:** 2026-04-28

S. He, T. Hou, J. Zhou, et al., “Endothelial Cells Promote Migration of Mesenchymal Stem Cells via PDGF‐BB/PDGFRβ‐Src‐Akt in the Context of Inflammatory Microenvironment upon Bone Defect,” *Stem Cells International*, 2022, 2401693, https://doi.org/10.1155/2022/2401693.

In the article, the authors have identified errors in Figure 1. Specifically, in Figure 1b, there are repeated elements between the JNJ‐10198409 and shPDGF‐BB groups.

**Figure 1:**
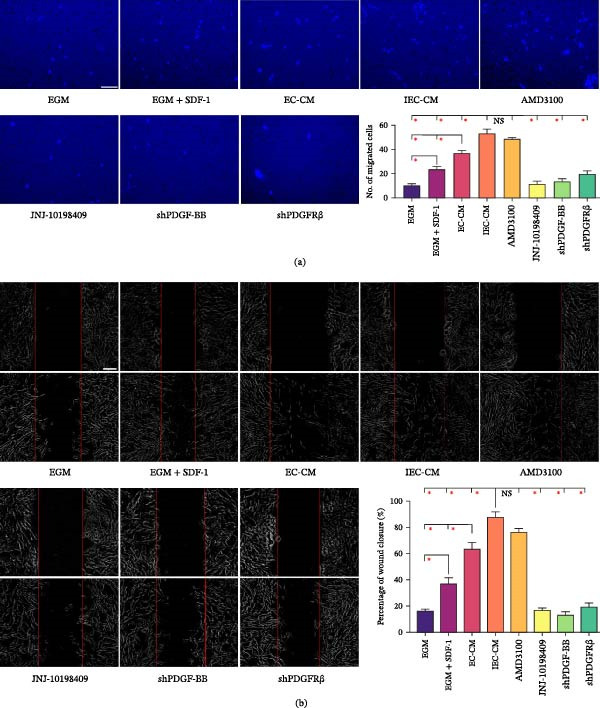
MSCs migrated toward ECs via PDGF‐BB/PDGFRβ in the inflammatory microenvironment. (a) Representative images of migrated hBMSCs that had received different pre‐treatments or had been exposed to different inducing media. The migration capacity of hBMSCs was determined using a Transwell culture system. The quantification of migrated cells was shown as a bar graph. Scale bar, 50 μm.  ^∗^ *p* < 0.05. (b) Representative images of wound healing assays. The rate of scratch wound closure was shown as a bar graph. Scale bar, 200 μm.  ^∗^ *p* < 0.05. EGM: endothelial cell growth medium‐2. EC‐CM: conditioned media of endothelial cells. IEC‐CM: conditioned media of endothelial cells in the context of inflammatory microenvironment; shPDGF‐BB: short hairpin RNA targeting *pdgfb*; shPDGFRβ: shRNA targeting *pdgfrb*.

After assessment by the editorial board of the authors’ request, they have confirmed that the results and conclusions are unaffected. The correct Figure 1 is shown below:

We apologize for this error.

