# Supplementary material for: Endothelial Cells Promote Migration of Mesenchymal Stem Cells via PDGF-BB/PDGFRβ-Src-Akt in the Context of Inflammatory Microenvironment upon Bone Defect
Source: Stem Cells Int. 2022 Sep 24;2022:2401693. doi: 10.1155/2022/2401693 (PMC9526552; doi:10.1155/2022/2401693)
Supplement: Supplementary Materials — Supplementary Figure 1. Scheme illustration for in vivo experiments. Femoral critical-sized bone defects were created in C57 mice and DBM were implanted. Implants harvested at postoperative day 1, 3, and 7 were subjected to fluorescence-activated cell sorting (FACs), RT-PCR, and western blot. At 7 days, wild GFP+ mBMSCs or cells intervened by shRNA were injected via tail vein every 2 days. Implants harvested at 10 days and 4 weeks were subjected to immunofluorescence and HE&Masson staining, respectively. Supplementary Figure 2. (A) ELISA results. (B) Interference efficiency of shRNA targeting pdgfb in HUVECs. (C) Interference efficiencies of shRNA targeting pdgfrb, src, and akt in hBMSCs. (D) Interference efficiencies of shRNA targeting pdgfrb, src, and akt in mBMSCs. Supplementary Figure 3. Representative images of H&E staining and Masson staining. At 4 weeks postoperatively, bony development was advanced within control group, as the implants were surrounded by chondrocyte, osteoblast-like cells, and filled with livable osteocytes. In the other groups, no viable osteocytes were found in lacunas within bone pieces, and implants were poorly embedded by osteogenesis-related cells. Scale bars, 500 μm. [file 2401693.f1.docx]

**
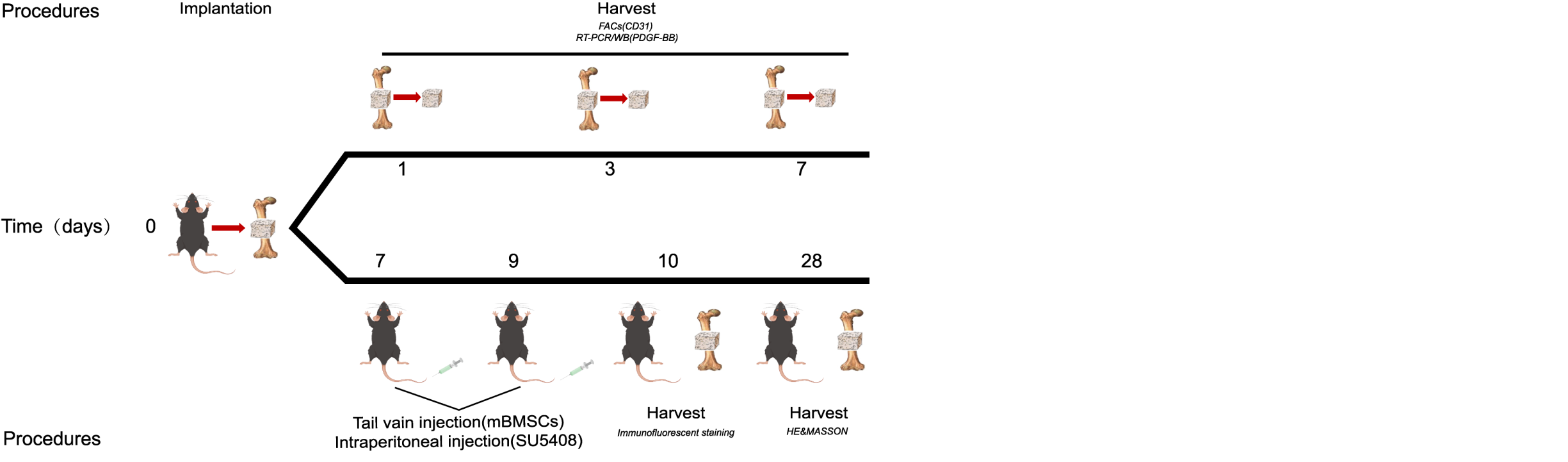
**

**Supplementary Figure 1.** Scheme illustration for *in-vivo* experiments. Femoral critical-sized bone defects were created in C57 mice and DBM were implanted. Implants harvested at postoperative day 1, 3, and 7 were subjected to fluorescence-activated cell sorting (FACs), RT-PCR and western blot. At 7 days, wild GFP^+^ mBMSCs or cells intervened by shRNA were injected via tail vein every 2 days. Implants harvested at 10 days and 4 weeks were subjected to immunofluorescence and HE&Masson staining, respectively.


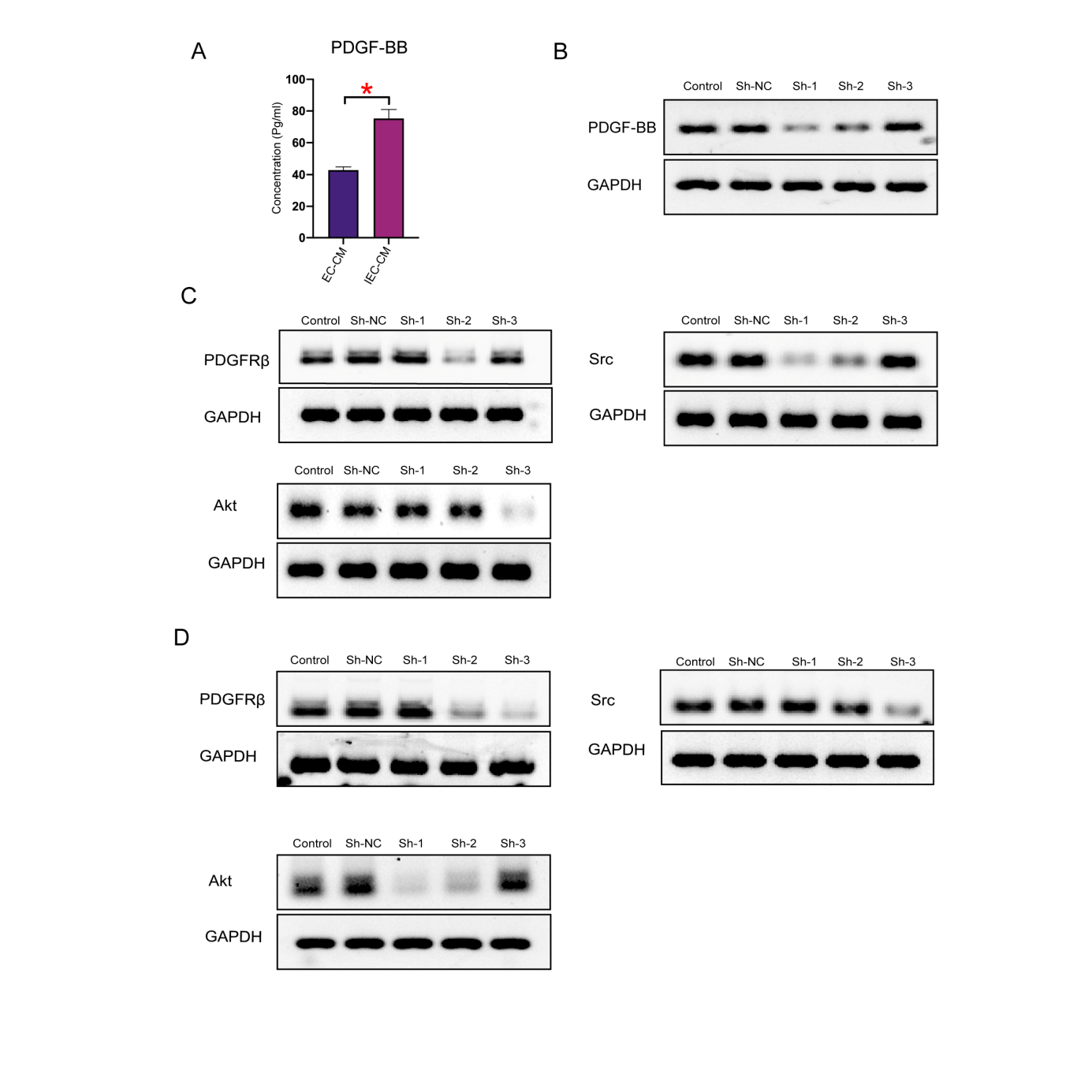


**Supplementary Figure 2.** (A) ELISA results. (B) Interference efficiency of shRNA targeting *pdgfb* in HUVECs. (C) Interference efficiencies of shRNA targeting *pdgfrb*, *src* and *akt* in hBMSCs. (D) Interference efficiencies of shRNA targeting *pdgfrb*, *src* and *akt* in mBMSCs


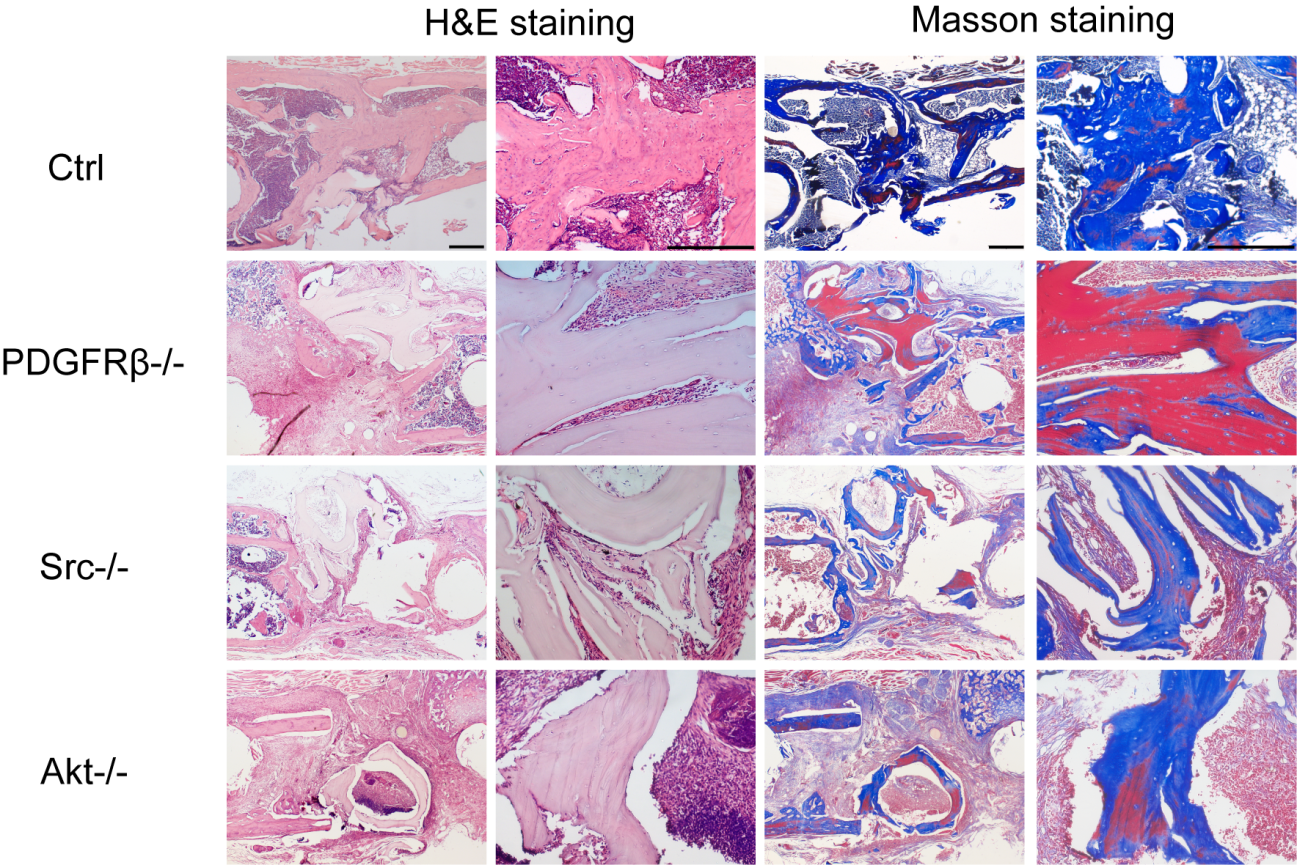


**Supplementary Figure 3.** Representative images of H&E staining and Masson staining. At 4 weeks postoperatively, bony development was advanced within control group, as the implants were surrounded by chondrocyte, osteoblast-like cells and filled with livable osteocytes. In the other groups, no viable osteocytes were found in lacunas within bone pieces, and implants were poorly embedded by osteogenesis-related cells. Scale bars, 500 μm.
